# Effect of L-theanine in the prevention of 2,4- dinitrochlorobenzene induced atopic dermatitis: A study in BALB/c mice model

**DOI:** 10.1016/j.toxrep.2025.101984

**Published:** 2025-03-01

**Authors:** Rajarshi Chattopadhyay, Souptik Iswar, Subhadeep Chakrabartti, Sayan Mondal, Preetam Modak, Pronabesh Ghosh, Saikat Mukherjee, Sumit Ghosh, Arindam Bhattacharyya, Avijit Dey, Parames C. Sil

**Affiliations:** a Department of Zoology, Ramakrishna Mission Vidyamandira, Belur Math, Howrah, West Bengal 711202, India; b Immunology Laboratory, Department of Zoology, University of Calcutta, 35, Ballygunge Circular Road, Kolkata, West Bengal 700019, India; c Boston Children’s Hospital, Harvard Medical School, 300, Longwood Ave, Boston, MA 02115, United States; d Division of Molecular Medicine, Bose Institute, P-1/12, CIT Scheme VII M, Kolkata, West Bengal 700054, India

**Keywords:** L-theanine, 2,4 dinitrochlorobenzene (DNCB), Filaggrin, Atopic dermatitis

## Abstract

Literature suggests that tea (*Camellia sinensis*) is a potent therapeutic agent and acts against many skin diseases. L-theanine, also known as γ-glutamyl ethylamide, is a non-protein amino acid, naturally obtained from tea and has structural similarity with the neurotransmitter glutamate. This study was conducted to evaluate the role of L-theanine, in the prevention of 2,4-dinitrochlorobenzene (DNCB) mediated atopic dermatitis (AD) in BALB/c mice. To achieve our goal, serological, histological, immunohistochemical and behavioural analyses, biochemical assays, immunophenotyping, skin scoring, etc. were carried out. L-theanine and DNCB docking with filaggrin protein was also checked as a part of *in-silico* analysis. No significant changes in serological profile were found, however, a substantial rise in leukocytes and macrophages were observed in the DNCB administered animals. L-theanine treatment altered such effects. ProTox profiling of L-theanine didn’t show cytotoxicity. No significant changes were observed in any other profiling assays except in the skin histology. Further, L-theanine was found to bind more effectively at the active site of filaggrin (key protein affected in AD) than DNCB. Therefore, L-theanine represents itself as a potent ameliorative agent against DNCB induced atopic dermatitis.

## Highlights

• L-theanine exhibits potential skin wound healing properties.
• L-theanine exerts anti-apoptotic effects.
• L-theanine binds more effectively at the active site of filaggrin than DNCB.
• L-theanine is as a potent ameliorative agent against DNCB induced AD.

## Graphical Abstract

**Figure:**
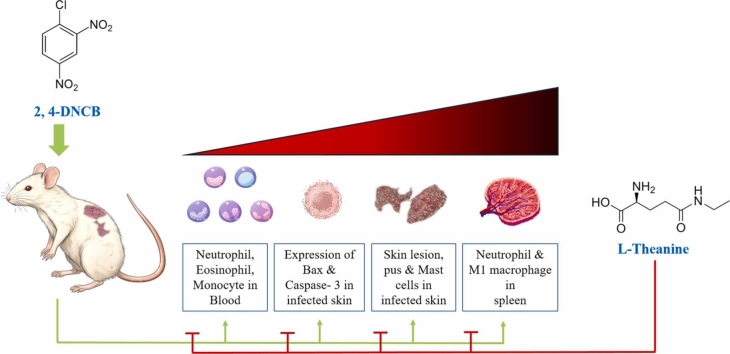

## 1 Introduction

Atopic dermatitis (AD) is a prevalent, long-lasting, inflammation associated skin condition which distresses 1 % of adults and up to 20 % of children globally. The genesis of this pathophysilogy is complicated, involving pathways of inflammation and immunity [1], [2]. It is commonly characterized by dry scaly skin, redness, swelling, intense itching and the appearance of lumps or rashes on the skin. Relapsing eczema with severe xerotic skin pruritus is a hallmark of AD. Immunosuppressive medications, including corticosteroids, methotrexate, cyclosporine, tacrolimus and pimecrolimus, have been extensively utilized to temporarily manage AD symptoms [3], [4], [5], [6], [7]. Nevertheless, skin atrophy, osteoporosis, skin cancer and metabolic problems are among the unfavourable side-effects of these medications. Finding substitute treatments that can lessen the effects of atopic illness is therefore essential. Literature also suggests that the genetical background of AD is linked to filaggrin (FLG) [5], [6], [7]. People with certain loss of function mutations of filaggrin are found to have a resilient genetic predisposition for atopic dermatitis [8], [9].

Various bioactive phytochemicals have been investigated for their therapeutic potential [10], [11], [12], [13], [14], [15], [16], [17]. Amongst them, L-theanine is a primary component of tea (*Camellia sinensis*), predominantly known for its neurological and cognitive benefits. It is a non-proteinogenic amino acid derived from γ-N-ethyl glutamine [18]. Its molecular formula is C_7_H_14_N_2_O_3_, which is also somewhat structurally similar to glutamate, thus mediating neurotransmitter functioning in the brain. It influences soothing effects, brain wave activity and increases alpha wave production, thereby promoting relaxed alertness. Its role in production of dopamine and serotonin helps it in those modulations [19]. L-theanine houses an array of different physiological and pharmacological benefits which showcase its role in stress reduction, immune modulation, cognitive boosting, oncological study, neuroprotection etc., making it a keystone in a large number of research fields. Its role in modulations of neurotransmitters like serotonin and dopamine helps in curbing stress and anxiety. Reducing oxidative stress and inhibiting cognitive impairments in animal models have been achieved by using L-theanine [20]. L-theanine may help to reduce inflammation and skin irritation on atopic dermatitis. L-theanine possesses antioxidant properties which may protect the skin from free radical induced oxidative stress [21], [22], [23], [24]. Additionally, it has been noted that L-theanine reduces the acute skin irritation caused by 2-O-tetradecanoylphorbol-13-acetate (TPA), diminishes inflammation of skin due to psoriasis and drastically lowers IL-23 and other chemokine levels [25]. In spite of being used in multiple research fields, the detailed effects of L-theanine on AD is yet to be pondered upon. Research on direct interactions of L-theanine with the skin barrier is very limited. Moreover, the field of study involving the anti-inflammatory properties of L-theanine in treating the inflamed and immune dysregulated environment of dermatitis is highly unexplored [26].

To assess medication candidates, epicutaneous sensitization to irritants like 2,4 dinitrochlorobenzene (DNCB) is a popular method used to induce AD due to the complex and significant aspects related to the pathophysiology of the disease. In this study, DNCB mediated allergic contact dermatitis (ACD) model was generated in BALB/c mice to simulate symptoms of AD and the potential ameliorative effects of L-theanine in mitigating the symptoms of AD was investigated.

## 2 Materials and methods

### 2.1 Chemicals

2,4-dinitrochlorobenzene, L-theanine (SRL, India), potassium phosphate monobasic, potassium phosphate dibasic, DPX, N-(1-naphthyl)-ethylenediamine dihydrochloride, sulfanilic acid, sodium nitrite, picric acid, oxo-glutaric acid, chloroform, RBC lysis buffer, alpha-ketoglutarate and aspartic acid have been used in this study. TRIzol Reagent (Ambion, by Life technologies, USA), eosin, Drabkin’s solution (Sigma, USA), ethanol, methanol, formaldehyde solution, hydrogen peroxide, paraffin wax, potassium hydroxide, sodium pyruvate, xylene (Merck, India), hematoxylin (Merck, Germany), glycerol anhydrous, glacial acetic acid (Merck-Millipore, India), sodium chloride, sodium di-hydrogen phosphate (SRL, India) were also used. All chemical reagents used were of molecular grade.

### 2.2 Experimental animals

BALB/c mice (24 ± 2 g) were housed in polypropylene mice cage VT/PP/290/MC (290 ×220 ×140 mm) with optimum conditions (12 h light and dark cycle), controlled temperature (24 ± 2°C) and relative humidity of about 65 %. The animals were subjected to standard pellet diet, vegetables and water *ad libitum*. They were acclimatized to laboratory conditions for a period of 14 days. The animal handling and experiments were performed at Bose Institute, India, following the guidelines of the IAEC (Institutional Animal Ethical Committee). The study was authorized by IAEC, CPCSEA (Committee for the Purpose of Control & Supervision on Experiments on Animals), and the Ministry of Environment and Forests, Govt. of India, New Delhi, India [1796/GO/EReBiBt/S/14/CPCSEA]. Four experimental groups with 6 mice in each group were segregated. Control with no treatment, AD where DNCB was administered and AD + T (50 mg/kg) & AD + T (100 mg/kg) were the groups where AD was induced along with L-theanine treatment of 50 mg/kg and 100 mg/kg body weight respectively.

### 2.3 Removal of dorsal hair and administration of DNCB

Skin on the dorsal side of BALB/c mice was targeted and sensitized with DNCB to develop AD-like symptoms [27]. Acetone and olive oil are mixed in 3:1 ratio and used as solvent to prepare DNCB solutions. 150 μL of 2 % DNCB solution was rubbed on the dorsal skin (in an area of 4 cm^2^) on day 0. From the 4th till 28th day, 150 μL of 0.5 % DNCB was applied to the same area in every three days to elicit AD-like symptoms [4].

### 2.4 Determination of toxicity and administration of L-theanine

**Fig. 1:**
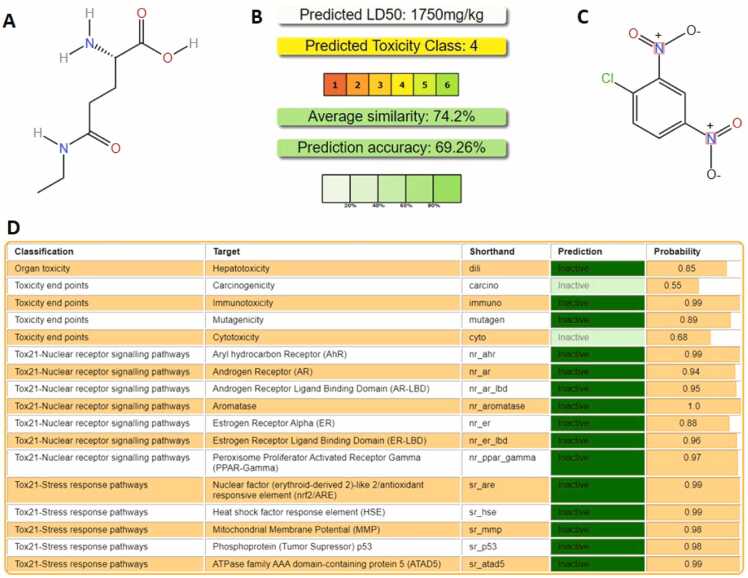
Estimation of Toxicity using ProTox II: (A) Structure of L-theanine, (B) Predicted LD_50_ value of L-theanine, Toxicity class, average similarity and prediction accuracy has been summarily produced, (C) Structure of 2,4-dinitrochlorobenzene, (D) The classified toxicity against various targets, pathways or stresses have been tabulated.

A Toxicity test for L-theanine using ProTox-II - Prediction of Toxicity of Chemicals, ProTox-II - Prediction of TOXicity of chemicals (charite.de) was conducted. With respect to Fig. 1, two doses of L-theanine at 50 mg/kg and 100 mg/kg body weight were selected [28], [29], [30], [31], [32].

### 2.5 *In vivo* experimental design

**Fig. 2:**
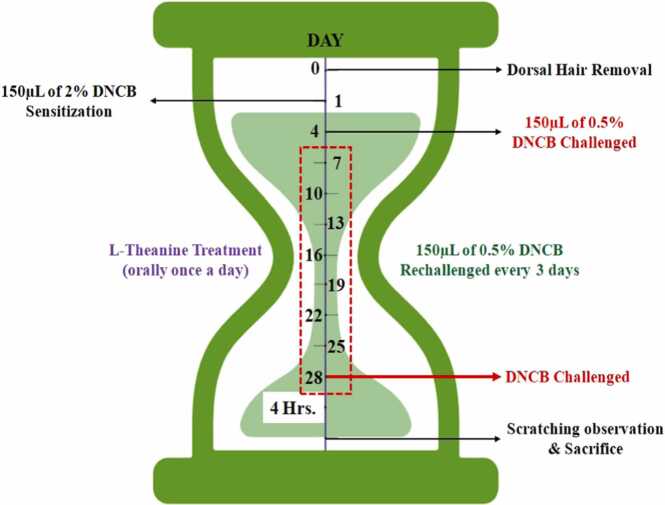
*In vivo* experimental design. BALB/c mice with removed dorsal hair were sensitized with 2 % DNCB on day 1, followed by repetitive 0.5 % DNCB challenges on every third day. Additionally, 50 and 100 mg/kg body weight of L-theanine were orally administered daily from day 7 to day 28, before the mice were euthanized 4 h after the final DNCB challenge. The pathological conditions resulting from DNCB irritation, with or without L-theanine treatment, were then examined.

On day 0 after initial weight measurement, 2 % DNCB was applied to all mice of groups AD, AD + T (50 mg/kg) & AD + T (100 mg/kg) over the exposed skin on dorsal side. On day 1, 0.5 % DNCB solution was added to mice in groups AD, AD + T (50 mg/kg) & AD + T (100 mg/kg) and DNCB solvent was applied to the group Control. Comparative observations were made and behavioural tests were performed 4 h after DNCB application. Starting from day 1, after every three days, 0.5 % DNCB solution was applied to the 3 groups. L-theanine was dissolved in 0.87 % saline to prepare the dosages of 50 mg/kg and 100 mg/kg body weight and given orally to AD + T (50 mg/kg) & AD + T (100 mg/kg) groups once daily using oral gavage, from day 7 till day 27. On the 28th day, skin scoring was done and mice were euthanized. Blood was collected from the heart and vital organs were collected for serological and histological studies (Fig. 2).

### 2.6 Measurement of body weight

The body weight of mice of different groups was noted in every 7 days starting from day 0 to day 28.

### 2.7 Scoring the epidermal site of AD induction and estimation of the severity index

The epidermal study and scoring of the AD sites have been based on previous works [33], [34], [35].

### 2.8 Study of locomotory activity

The mice were subjected to open field test. The locomotive activity of mice was studied in an open field box. Locomotion was recorded for two min.

### 2.9 Collection of blood

Blood was collected by cardiac puncture from 4 different experimental groups. Blood was aliquoted in heparinized vials (for TC RBC, DC WBC). Sera were prepared from another aliquot of blood.

### 2.10 Study of haematological profile

#### 2.10.1 Estimation of haemoglobin

Drabkin's solution (5 ml) was added to a test tube. Following a gentle inversion of the blood sample, 20 µl of heparinized blood was taken into a pipette. To get rid of extra blood, the outside of the pipette was cleaned. Blood was gradually released into Drabkin's solution using a pipette that was inserted into the tube. Following a thorough mixing, it was incubated for 5 min and absorbance was measured in a spectrophotometer (Shimadzu, Japan) at 540 nm [36].

#### 2.10.2 Estimation of total RBC count

10 µl of blood was poured in 4 ml RBC diluting fluid and mixed carefully. 10 µl of it was added to the haemocytometer’s top and bottom counting chambers, left for 3 min to allow the cells to settle. Total number of RBCs was recorded in 400X magnification under bright field light microscope [37].

#### 2.10.3 Estimation of total WBC count

50 µl of blood was poured in 950 µl WBC diluting fluid and mixed carefully. 10 µl of it was added to the haemocytometer’s top and bottom counting chambers, left for 3 min to allow the cells to settle. Total number of WBCs was recorded in 400X magnification [37].

#### 2.10.4 Estimation of differential count of WBC

Thin blood films were prepared on slides and fixed with methanol. They were stained with Giemsa’s stain for 45 min. The blood films were then observed under bright field microscope (Olympus, Japan) at 400X. A total number of five hundred WBCs were counted from different fields and percentage of lymphocyte, neutrophil, monocyte, eosinophil and basophil were calculated.

### 2.11 Study of serological profile

#### 2.11.1 Estimation of nitrite (NO)

The generation of NO was measured with Griess reagent [38]. 200 µl serum mixed with equal volume of Griess reagent (0.1 % naphthyl ethylenediamine dihydrochloride, 1 % sulphanilamide and 5 % orthophosphoric acid). The mixture was incubated for 30 min at 37°C in a humid chamber. The absorbance was recorded at 550 nm in spectrophotometer (Shimadzu, Japan).

### 2.12 Study of liver function

To evaluate liver function, glutamic pyruvic transaminase (SGPT) or alanine aminotransferase (ALT) and serum glutamic oxaloacetic transaminase (SGOT) or aspartate aminotransferase (AST) levels were measured from serum following Reitman & Frankel’s Method [39]. In a spectrophotometer, the absorbance was measured at 505 nm after 10 minutes. Three times the complete experiment was conducted in analogous settings. A pyruvate standard curve was used for further calculation.

### 2.13 Histological sample preparation

The liver and skin were taken from the euthanized mice and thin sections were made using microtome following which HE staining was performed. The tissue sample were analysed using Olympus BX43 Upright Microscope.

### 2.14 Immunohistochemistry

10 % neutral buffered formalin was used to fix tissue samples, subjected to graded alcohol dehydration and embedded in paraffin. 5 µm tissue section was prepared using microtome, taken in slide, deparaffinised and rehydrated with down grade of ethanol. Then the sections were incubated with primary antibodies against Bax, Bcl-2 & Caspase 3 (Santa Cruz, CA, USA) and were incubated with a HRP tagged secondary antibody. DAB detection kit was used for colorimetric detection of antibody binding sites, followed by hematoxylin counter staining [40].

### 2.15 Mast cell staining

The skin samples, taken from the euthanized mice, were paraffinized and thin sections were made using microtome. The sections were deparaffinized with xylene and hydrated with ethanol downgradation and finally dipped in distilled water. The sections were then stained with toluidine blue (1 % toluidine blue in 70 % ethanol, 5 ml; 1 % sodium chloride aqueous, 45 ml; pH adjusted to 2.5) and observed under a light microscope. Mast cells appeared violet against bluish background.

### 2.16 Flow cytometry analysis

Spleens were collected aseptically from all experimental groups post euthanasia. Immediately single-cell suspensions were generated from whole spleens and were passed through a cell strainer. Single-cell suspensions were incubated with lysis buffer for 5 min to remove the RBCs. The suspension was washed with cold DMEM and the splenocytes were resuspended in FACS buffer and then incubated for about 30–40 min on ice with fluorochrome-conjugated antibodies (PerCP-Cy5.5 Anti-CD11b, FITC Anti-Gr1, APC Anti-F4/80, anti CD206, Anti-CD80 PE etc) for the appropriate isotype controls. Data were then acquired using BD FACSAria III and analyzed using Flowjo software.

### 2.17 Docking using AutoDock/Vina

AutoDock Tools (ADT) were used to assign polar hydrogens and unified atom Kollman charges to the protein. AutoDock was used to save the prepared file in PDBQT format. Using a grid box, AutoGrid was used to prepare the grid map and the grid centre was assigned at the XYZ dimensions. Grid box properties, protein and ligand information and AutoDock/Vina were used for docking in the conformation files. Iterated local search global optimizer was used in AutoDock/Vina [41]. The position with the lowest binding energy was matched with the structure of the receptor.

### 2.18 Preparation of scientific diagrams and chemical structures

KingDraw was used for the preparation of chemical structures.

### 2.19 Prediction of drug-likeness

The ADMET analysis and evaluation of drug likeness was performed using Swiss ADME (developed by Swiss Institute of Bioinformatics) availed at www.swissadme.ch and the Lipinski’s filter was used to analyse drug likeness [42], [43].

### 2.20 Statistical analysis

Various statistical analyses were employed, contingent on the quantity of groups, nature of data, data distribution, and experiment type. Both parametric & non-parametric tests were performed in the case of continuous data based on their distribution. For every experiment conducted with two samples, either the non-parametric Mann-Whitney test or the parametric student's *t*-test was performed. Post hoc Dunn tests for multiple comparisons using non-parametric Kruskall Wallis ANOVA have been conducted. Version 6.0 of the GraphPad Prism program was used. Bar graphs use the mean ± standard error of mean (SEM) to display data. P values less than 0.05 (p < 0.05) were regarded as significant.

## 3 Results

### 3.1 Effect on body weight

**Fig. 3:**
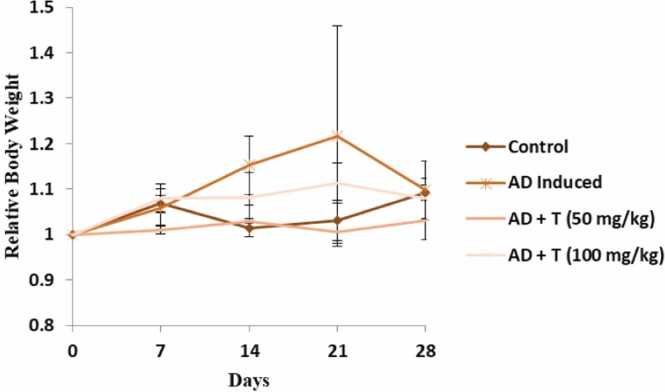
Relative weight of mice of all experimental groups. Data represent the variation of weight with respect to that observed on the first day, on specific intervals. Data in bar diagrams are represented as mean ± standard error of mean (Mean ± SEM) [Level of significance: ns: non-significant, *P < 0.05, **P < 0.01, ***P < 0.001, ****P < 0.0001].

The body weights of the experimental animals showed minor variations in comparison to the control, with respect to that observed at the beginning of the experiments (Fig. 3). This implies that, within the parameters of this experiment, L-theanine at 50 mg/kg body weight may not significantly influence the overall weight of the animal body. The group wise and day wise variation of the body weight were found to be non-significant.

### 3.2 Effect on weight and size of vital organs

**Fig. 4:**
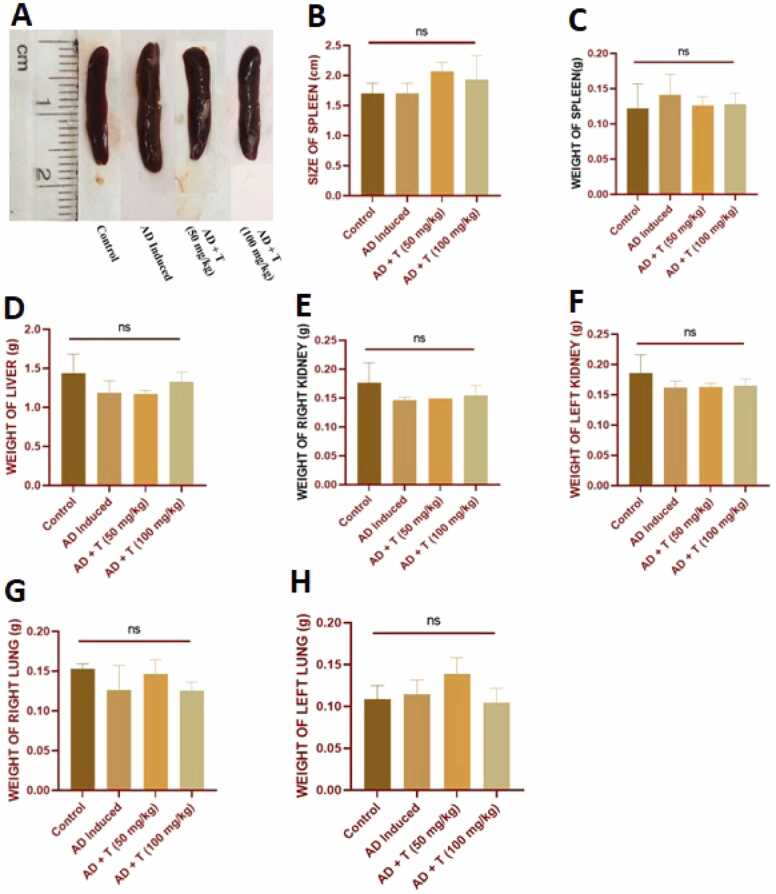
The data in the figure represents: (A-C) splenic dimensions in experimental mice, (D) the mean weight of liver from all the treatment groups at 28th day, (E, F) mean weight of right and left kidney from all the treatment groups, at 28th day, (G, H) mean weight of right and left lungs from all the treatment groups, at 28th day. Data in bar diagrams are represented as Mean ± SEM [Level of significance: ns: non-significant, *P < 0.05, **P < 0.01, ***P < 0.001, ****P < 0.0001].

No significant alteration was observed in the splenic dimensions among animals of all the experimental groups (Fig. 4**A-C**). The weight of the vital organs (liver, kidney, spleen and lungs) of the experimental animals of the four groups was not found to be significantly altered (Fig. 4**D-H**). This implies that, within the parameters of this experiment, L-theanine at these doses may not significantly influence the weight and size of the vital organs in the context of atopic dermatitis treatment.

### 3.3 Effect on locomotory activity

**Fig. 5:**
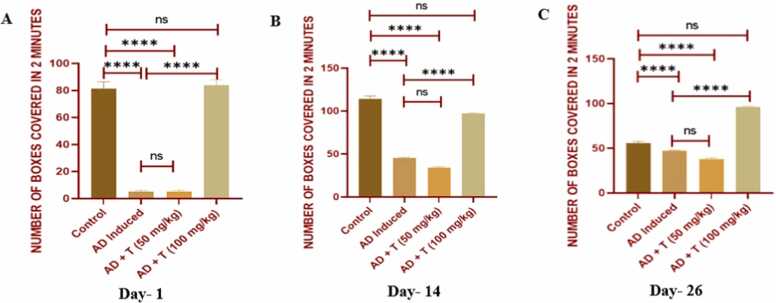
The result of behaviour analysis: (A) On day 1 of the experiment, (B) On day 14 of the experiment, (C) On the day 26 of the experiment. Data in bar diagrams are represented as Mean ± SEM [Level of significance: ns: non-significant, *P < 0.05, **P < 0.01, ***P < 0.001, ****P < 0.0001].

Foraging activity was found to be low in the AD group on day 1 compared to the control group. A significantly high foraging activity was observed in the AD + T 100 mg/kg group on day 1, 14 & 26 compared to the AD group (Fig. 5).

### 3.4 Effect on epidermal site of atopic dermatitis induction

**Fig. 6:**
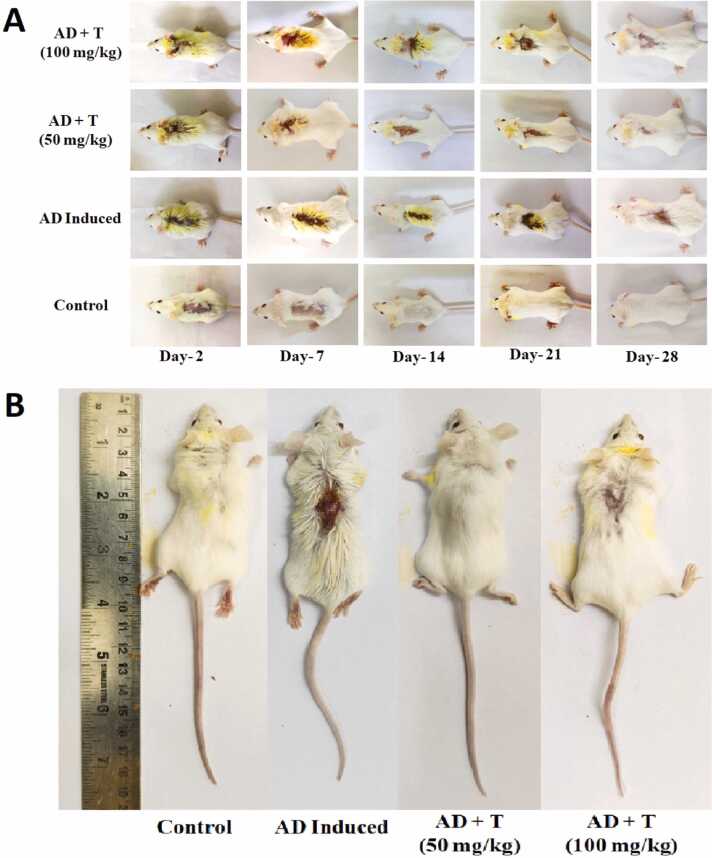
Effect of DNCB and L-theanine on different treatment groups: (A) the comparative effect on the treatment groups at regular intervals, the curative effect of 50 mg/kg and 100 mg/kg body weight L-theanine doses can be observed (B) the comparative effect on exposed dermal site at 28th day, before euthanized.

The epidermal site of AD induction was seen to get cured with L-theanine. It has been observed that the ameliorative effect of L-theanine seems more effective at 50 mg/kg than 100 mg/kg body weight (Fig. 6 **A, B**). The severity index of AD seems maximum in the AD group. The severity index of AD + T (50 mg/kg) stands minimal, suggesting effective therapeutic efficacy of L-theanine at the said dose when used against AD (Table 1). The groups were further assessed in accordance to the scoring scale of International Contact Dermatitis Research Group (ICDRG), and the disease group were acknowledged with maximum score (+++), followed by a doubtful scoring (+?) against AD + T (100 mg/kg). Moreover, the length of lesion, the scratching numbers (in 2 min) and the Romanian studies were conducted at the AD site on the category of lesion. They suggest the dose specific therapeutic effect of L-theanine on the AD + T groups (Table 2).

**Table 1:** Severity Indexing based on scoring of length of lesion, scratching score and grade of dermatitis. *Control: no treatment, AD: DNCB was administered, AD + T (50 mg/kg) & AD + T (100 mg/kg): AD induced along with L-theanine treatment of 50 mg/kg and 100 mg/kg body weight respectively.*

***Groups***	***Lesion Length Score (0−3) [L]***	***Scratching Score (0−3) [S]***	***Dermatitis Grade (0−4) [D]***	***Severity Index [(L+S+D)/10]***
Control	0	0	0	0
AD Induced	3	3	4	1
AD + T (50 mg/kg)	0	0	0	0
AD + T (100 mg/kg)	1	1	1	0.3

**Table 2:** Romanian Studies of skin with other morphological parameters of AD with The International Contact Dermatitis Research Group (ICDRG) Scoring. *Control: no treatment, AD: DNCB was administered, AD + T (50 mg/kg) & AD + T (100 mg/kg): AD induced along with L-theanine treatment of 50 mg/kg and 100 mg/kg body weight respectively.*

***Groups***	***Length of Lesion (cm)***	***Scratching Number*** ***(in 2 min)***	***Appearance at skin site: Romanian Studies***	***ICDRG Scoring Scale***
Control	0	0	No lesion present	***-***
AD Induced	2.6	30–35	Severe (deep red erythema over entire site with excoriations)	***+++***
AD + T (50 mg/kg)	0	0	Normal	***-***
AD + T (100 mg/kg)	0.6	2	Mild (slightly pink discoloration over most of site, no oedema)	***+*****?**

### 3.5 Effect on haematological profile

#### 3.5.1 Haemoglobin estimation

**Fig. 7:**
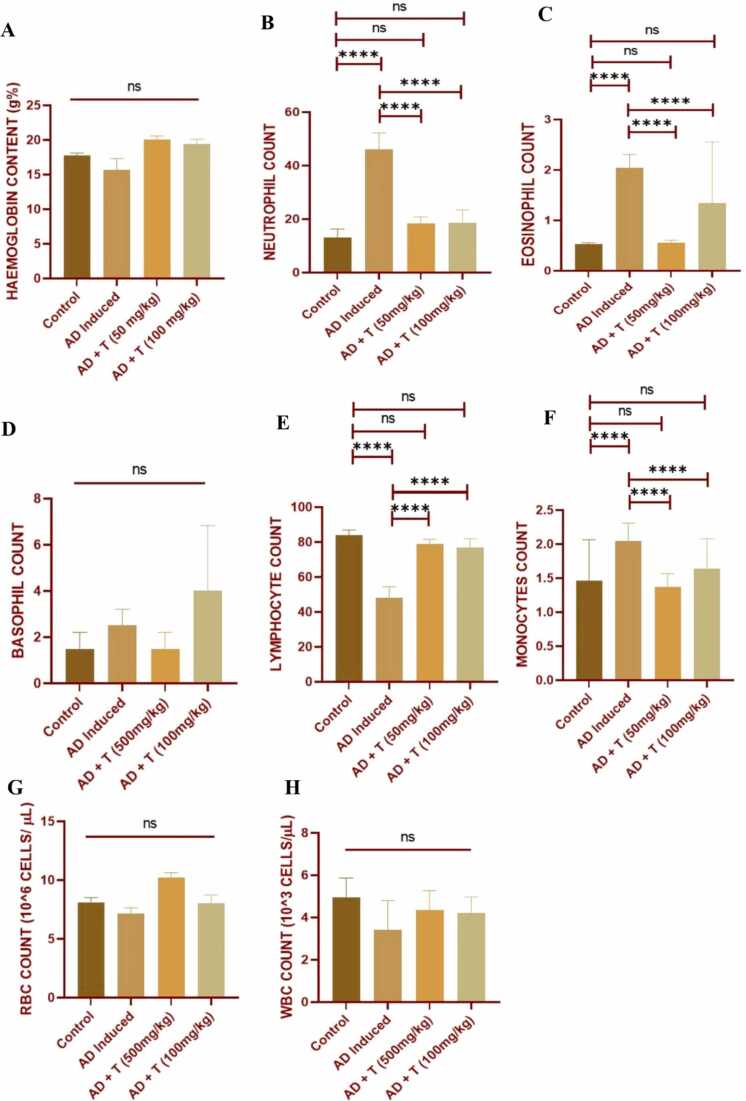
The bar graph represents: (A) haemoglobin content across all the treatment groups. The differential count of different types of WBCs as present in the control group in comparison to the AD and treatment groups, (B) neutrophil count, (C) eosinophil count, (D) basophil count, (E) lymphocyte count and (F) monocyte count. The total count (G) RBC, and (H) WBC across all the treatment groups in 28th day has been represented. Data in bar diagrams are represented as Mean ± SEM [Level of significance: ns: non-significant, *P < 0.05, **P < 0.01, ***P < 0.001, ****P < 0.0001].

The analysis reveals that after administration of 50 mg/kg and 100 mg/kg body weight of L-theanine orally to atopic dermatitis mice, the haemoglobin content exhibited no significant changes compared to the control and AD group. This indicates that, within the parameters of this experiment, L-theanine at these doses may not significantly influence the haemoglobin content in the context of atopic dermatitis treatment (Table 3**,** Fig. 7**A**).

**Table 3:** Haematological profile of different treatment groups (N- Neutrophils, E- Eosinophils, B- Basophils, L- Lymphocytes, M- Monocytes). TC: Total count, DC: Differential Count. *Control: no treatment, AD: DNCB was administered, AD + T (50 mg/kg) & AD + T (100 mg/kg): AD induced along with L-theanine treatment of 50 mg/kg and 100 mg/kg body weight respectively.*

***Treatment Groups***	***TC RBC (x10***^***6***^***)***	***TC WBC (x10***^***3***^***)***	***DC WBC (%)***
Control	7.4800	4.1500	N:16.751269; B:1.02564103; E:0.5714286; M:2.2857143; L:87.17948718
AD	7.1500	3.4250	N: 53.2544379; B: 1.72413793; E: 2.3668639; M: 2.3668639; L: 56.32183908
AD + T (50 mg/kg)	9.5575	3.1125	N: 20.5263158; B: 1.05263158; E: 0.6097561; M: 1.5789474; L: 81.87134503
AD + T (100 mg/kg)	7.5450	3.3125	N: 25.5102041; B: 2.91262136; E: 2.9126214; M: 1.9417476; L: 83.33333333

#### 3.5.2 Differential count of WBC

The analysis suggests that L-theanine administration has a potential mitigating effect on neutrophil, eosinophil, lymphocytes and monocytes infiltration associated with atopic dermatitis. L-theanine's anti-inflammatory impact, reflected in the reduced eosinophil recruitment, supports its role as a therapeutic agent in countering the immune response triggered by DNCB-induced atopic dermatitis. These findings emphasize the potential of L-theanine in modulating immune cell dynamics to alleviate inflammation in the context of skin diseases (Table 3**,** Fig. 7 **B-F**).

#### 3.5.3 Total count of RBC and WBC

The analysis reveals that after administration of 50 mg/kg and 100 mg/kg body weight of L-theanine orally to atopic dermatitis mice, the total number of RBCs and WBCs exhibited no significant changes compared to the AD group. It implies that, within the parameters of this experiment, L-theanine at these doses may not significantly influence the total number of RBCs and WBCs in the context of atopic dermatitis treatment (Fig. 7 **G, H**).

### 3.6 Effect on liver function

**Fig. 8:**
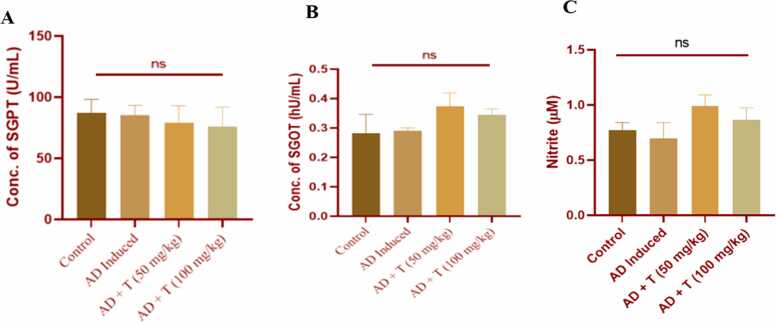
Concentration of (A) SGPT, (B) SGOT and (C) nitrite (NO). Data in bar diagrams are represented as Mean ± SEM [Level of significance: ns: non-significant, *P < 0.05, **P < 0.01, ***P < 0.001, ****P < 0.0001].

After 21 days of administering 50 mg/kg and 100 mg/kg body weight of L-theanine orally to atopic dermatitis mice, the SGPT and SGOT levels did not exhibit any significant change compared to the AD group (Fig. 8 **A, B**). This implies that, within the parameters of this experiment, L-theanine doses may not significantly influence liver function in the context of atopic dermatitis treatment.

### 3.7 Effect on nitrate generation

No statistically significant changes were also detected in the NO level (Fig. 8 **C**) of serum, among control, AD induced, AD + T (50 mg/kg) and AD + T (100 mg/kg) animals.

### 3.8 Histological changes

**Fig. 9:**
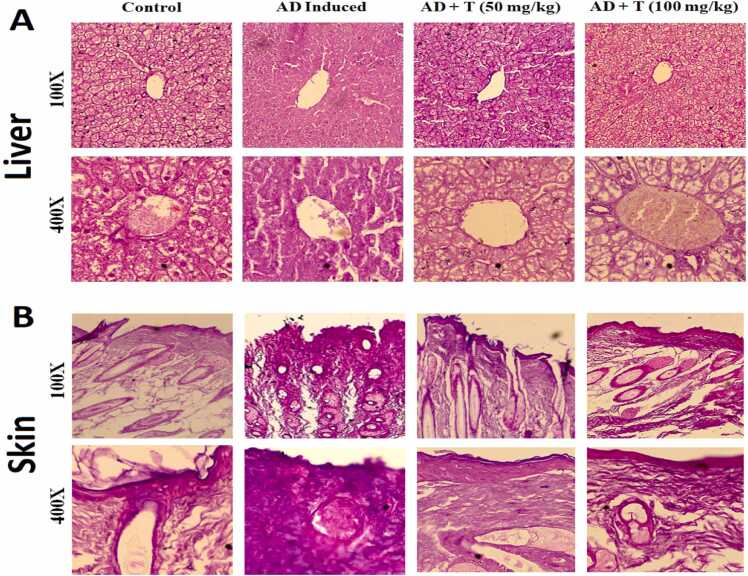
Histology of (A) Liver & (B) Skin after H&E staining; Magnification 100X and 400X on day 28.

No significant changes were observed in liver histology in either of the experimental groups. This suggests that L-theanine, has no effect in the liver (Fig. 9**A**). The AD group shows substantial inflammation in skin, with respect to control. The inflammation gets lowered in treatment group AD + T (50 mg/kg) more effectively than AD + T (100 mg/kg) (Fig. 9**B**). Result shows that L-theanine, effectively reduces DNCB induced AD, in a dose specific manner.

### 3.9 Analysis of the expression of apoptotic protein markers through immunohistochemistry

**Fig. 10:**
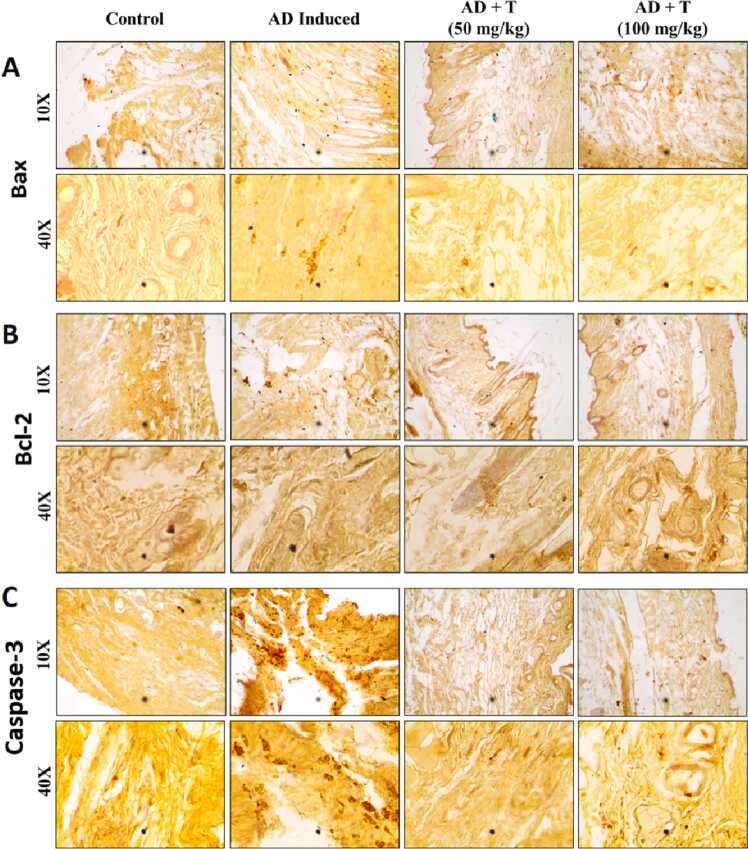
Observation of pro-apoptotic gene expression by immunohistochemistry: (A) Expression of Bax; (B) Expression of Bcl-II; (C) Expression of Caspase-III, prepared by adding Primary Antibody at 1:150 dilution followed by Secondary antibody at 1:200 dilution. The slides were subsequently stained with Delafield’s hematoxylin and were observed at 10X and 40X magnification.

Atopic dermatitis progresses due to the apoptosis of keratinocytes which results in the disintegration of the vesicular and epidermal structure [44], [45]. The expression of Bax, Bcl-2 and caspase 3 were investigated, as these proteins are implicated in the induction of apoptosis through intrinsic apoptosis pathways. Fig. 10 represents microscopic observations of the slides describing the significant differences between the dose specific ameliorative effect of L-theanine and the AD group. The results show the up-regulation of Bax and caspase-3 and down-regulation of Bcl-2 proteins in the AD group and subsequent alteration in the L-theanine treatment groups.

### 3.10 Analysis of mast cell infiltration

**Fig. 11:**
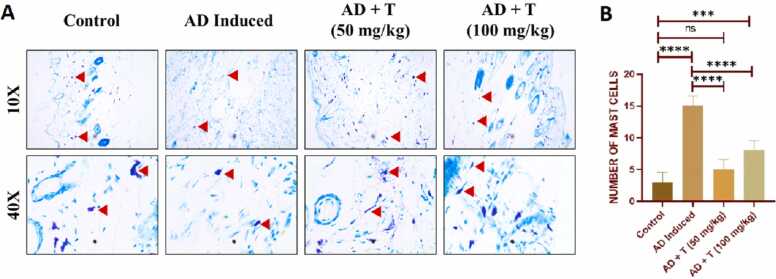
Mast cell infiltration observed by toluidine blue staining of the dermis. (A) qualitative measurement of mast cell numbers (marked with red arrow head); (B) quantitative measurement of mast cell numbers. Slides were examined at 10x & 40x magnification. Data in bar diagrams is represented as mean ± standard error of mean (SEM). Level of significance: ns: non-significant, *P < 0.05, **P < 0.01, ***P < 0.001, ****P < 0.0001.

In the AD mouse model, mast cell and lymphocyte infiltration are characterized by a thicker epidermal barrier and hypertrophy of the epithelial cells [46]. Thus, in mice treated with and without L-theanine, the epidermal thickness and the mast cell penetration into the dermis were measured. Comparing the mice exposed to repeated sensitization with DNCB to the normal mice, Fig. 11 illustrates the enhanced mast cell infiltration, epidermal thickness, and epidermal hyperplasia in the exposed mice. Nevertheless, when 50 or 100 mg/kg body weight of L-theanine was administered, the thickness of the epidermal region with hyperplasia and the mast cell count in the dermis decreased, supporting the idea that L-theanine has therapeutic effects in reducing AD-like symptoms.

### 3.11 Effect on neutrophils and macrophages

**Fig. 12:**
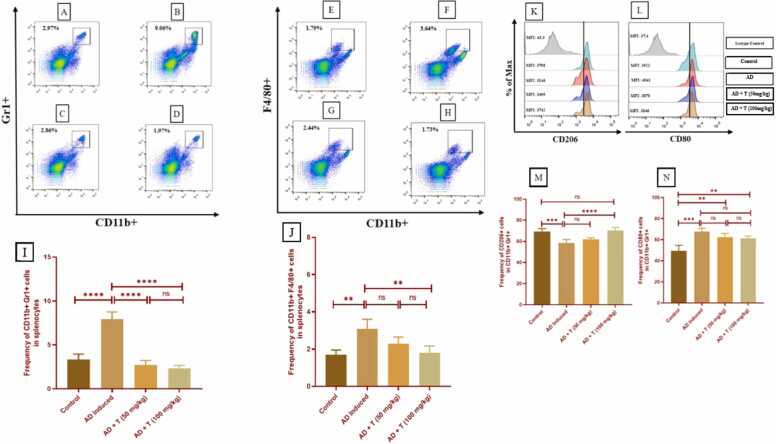
Flow cytometric analysis of spleen cells from experimental mice stained with CD11b+ and Gr-1 + antibodies is shown in the figure. Dot plots illustrate CD11b+ Gr-1 + cell percentages among total splenocytes in (A) Control, (B) AD, (C) AD + T (50 mg/kg), and (D) AD + T (100 mg/kg) groups. Bar diagrams (I) indicate CD11b+ Gr-1 + cell frequency in the spleen. Dot plots also display percentages of CD11b+ F4/80 + cells in (E) Control, (F) AD, (C) AD + T (50 mg/kg), and (D) AD + T (100 mg/kg), with bar diagrams (J) showing their frequency. Histograms and bar diagrams (K-N) present the percentage and frequency of CD206 + and CD80 + populations, with MFIs noted within. Gates were set based on isotype controls. Data is shown as Mean ± SEM, analyzed by Student's *t*-test, Mann-Whitney test, and ANOVA (ns: non-significant, *P < 0.05, **P < 0.01, ***P < 0.001, ****P < 0.0001).

Within spleenocytes, Gr1 + and CD11b+ populations, corresponding to neutrophils increases significantly by 9.06 % in AD group (Fig. 12 **B & I**) with respect to 2.97 % in Control (Fig. 12 **A**). However, in AD + T (50 mg/kg) and AD + T (100 mg/kg) the neutrophil population becomes significantly lower than AD (Fig. 12 **I**) and almost corresponds to Control, with 2.86 % and 1.97 % respectively (Fig. 12 **C & D**). Within spleenocytes, F4/80 + and CD11b+ populations, corresponding to macrophages, increases significantly by 3.64 % in AD group (Fig. 12 **F & J**) with respect to 1.79 % in Control (Fig. 12 **E**). However, in AD + T (50 mg/kg) macrophage population lowers to 2.44 % (Fig. 12 **G & J**), whereas in AD + T (100 mg/kg) it becomes significantly lower than AD and almost corresponds to Control, with 1.73 % (Fig. 12 **H & I**).

CD206 + population of M2 macrophages, within spleenocytes, seems to decrease substantially in AD group (Fig. 12 **K**), however, even though no significant hike in M1 macrophages is observed in AD + T (50 mg/kg), but, the normal population gets substantially replenished in AD + T (100 mg/kg) group, suggesting a significant hike the population of M2 Macrophages in AD + T (100 mg/kg) population with respect to AD (Fig. 12 **M**). To the contrast, CD80 + M1 macrophages, increases substantially in AD group, but no significant reduction is observed in AD + T (50 mg/kg) or AD + T (100 mg/kg) groups (Fig. 12 **L, N**). Thus, the neutrophil populations decreased substantially in the treatment groups, M1 macrophages increased significantly in the treatment groups with respect to the AD group, but no significant change in the M2 population was observed after treatment.

### 3.12 Analysis of molecular docking simulations

**Fig. 13:**
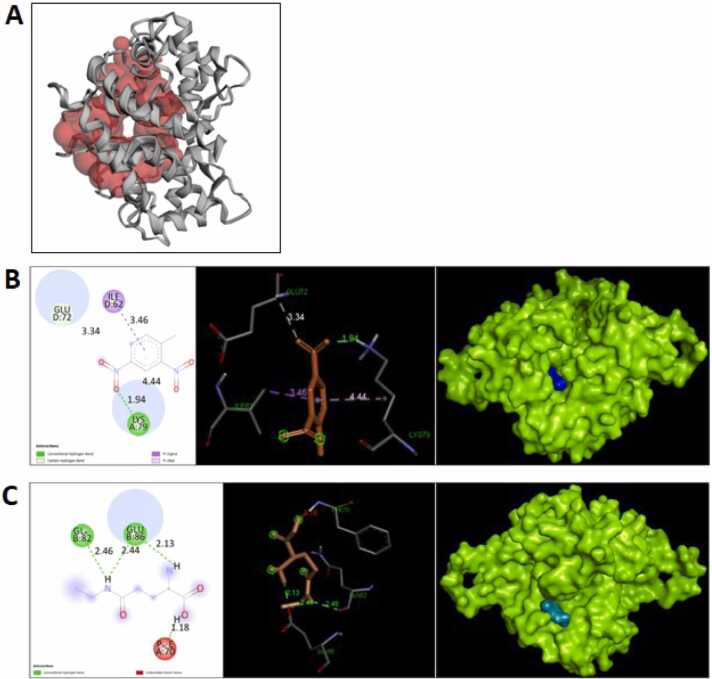
(A) Human Filaggrin active binding pockets were identified using CASTp, which employs Delaunay triangulation and Voronoi concepts to detect potential pockets and voids [53]. Protein-ligand interactions were simulated using AutoDock Vina: (B) DNCB with Filaggrin and (C) L-theanine with Filaggrin. Three-dimensional surface structures (AutoDock Vina) illustrate ligand binding pockets of receptor proteins in (A) and (B), while 2D representations highlight ligand interactions with specific amino acids in the protein chains.

Filaggrin helps to form the skin barrier and maintain the skin's hydration and pH [47], [48]. The binding energy values of protein-ligand complexes and the interacting amino acid residues have been provided (Fig. 13 **B&C;** Table 4 **&** 5). It was observed that L-theanine has better binding interactions with Filaggrin (-5 kcal/mol) than DNCB.

**Table 4:** Energy profile of binding interactions. *(DNCB: 2,4-Dinitrochlorobenzene)*.

Name of the ligand molecule	Affinity energy (kcal/mol)	Binding energy (kJ/mol)
L-theanine	−5	−3.02
DNCB	−1.7	−0.6

**Table 5:** List of interacting amino acid residues of the concerned receptor proteins. *(DNCB: 2,4-Dinitrochlorobenzene)*.

Name of the ligand molecule	Interacting amino acid residues of the receptor protein
L-theanine	Chain B: Glu−86 Chain B: Gln−82 Chain A: Phe−70
DNCB	Chain D: Ile- 62 Chain D: Glu- 72 Chain A: Lys- 79

**Fig. 14:**
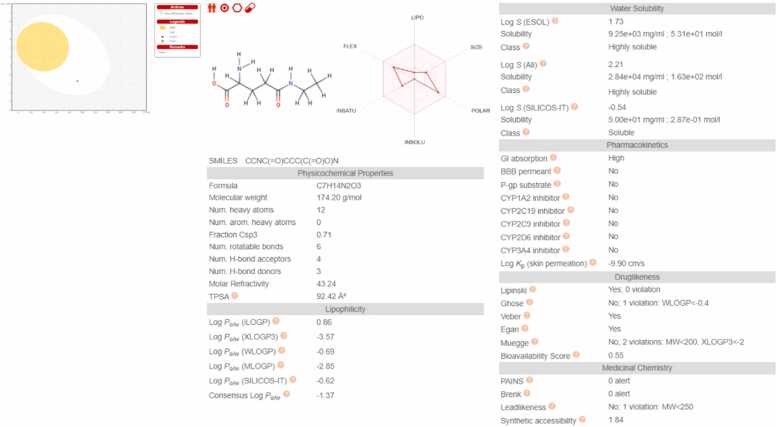
*In silico* analysis of L-theanine: Boiled egg structure & ADMET profile.

The ADMET (Absorption Distribution Metabolism Excretion and Toxicity) properties for the used drug molecule, L-theanine were analysed using Swiss ADME, which are demonstrated in Fig. 14. According to the Lipinski “Rule of 5” [49], L-theanine has a molecular weight 174.2 gmol^−1^ which is lower than 500 gmol^−1^. Further, it has less than 3H-bond donors and 4H-bond acceptors and has a molar refractivity between the range 40 and 130, which certifies it with 0 violations and predicting it as a safe molecule to be used for therapeutic purposes [50].

From the boiled egg structure [51] given in Fig. 14, it can be concluded that it is probably impermeable to the blood brain barrier but is actively absorbed by the gastrointestinal tract since it is not present in the yellow (yolk region) but present in the white (albumin region). These *in-silico* results indicate that L-theanine is safe to be used for human use.

## 4 Discussion

Owing to its physiological symptoms resembling those of AD, the DNCB sensitization is frequently used to evaluate the ameliorative effects of potential therapeutic compounds in mice. L-theanine has been taken for therapeutic consideration because of its low toxicity in mammalian cells and has been recognized as a powerful cancer prevention agent and calming specialist [21], [22]. In this study, L-theanine was orally administered in mice at doses of 50 mg/kg and 100 mg/kg body weight daily for 21 days. The dosages of L-theanine need to be further optimized in subsequent clinical studies. In this study, the potential therapeutic role of L-theanine against AD was surveyed. Oral administration of L-theanine has been partially cured the AD induced dermal site which is confirmed through histological analyses. It was observed that following L-theanine treatment, the pole cell hypertrophy and epidermal hyperplasia in the dermis were eased, the neutrophil populations decreased substantially, M1 macrophages increased significantly, but no significant change in the M2 population was observed. Apoptotic induction in the AD group was significantly reduced in the L-theanine treatment groups. Studies have shown that the expression of filaggrin decreases after DNCB administration [52]. Molecular docking simulation studies revealed that L-theanine has better binding interactions with FLG than DNCB. Therefore, these multitude of observations suggest that L-theanine can be a helpful up-comer.

## 5 Conclusion

During this study, we observed that mice treated with L-theanine with a dose of 50 mg/kg body weight showed better curative effects of L-theanine than ones with 100 mg/kg. So, we predict that the optimum dose for L-theanine must lie between 50 mg/kg – 100 mg/kg. This study holds promise to various gateways of further experimentation: (a) investigation of anti-inflammatory effects of L-theanine against AD through PCR amplification and immunophenotype analyses of the expression levels of different molecules in different tissues of the body, (b) special focus on the mechanism of protective effects of L-theanine, with minimal or no side effects.

## CRediT authorship contribution statement

**Mondal Sayan:** Writing – review & editing, Writing – original draft, Visualization, Validation, Investigation, Formal analysis. **Chakrabartti Subhadeep:** Writing – review & editing, Writing – original draft, Visualization, Investigation. **Iswar Souptik:** Writing – review & editing, Writing – original draft, Visualization, Methodology, Investigation, Formal analysis, Data curation, Conceptualization. **Chattopadhyay Rajarshi:** Writing – review & editing, Writing – original draft, Visualization, Methodology, Investigation, Formal analysis, Data curation, Conceptualization. **Sil Parames:** Writing – review & editing, Visualization, Validation, Supervision, Resources, Project administration, Methodology, Formal analysis, Data curation, Conceptualization. **Dey Avijit:** Writing – original draft, Validation, Supervision, Resources, Project administration, Methodology, Formal analysis, Data curation, Conceptualization. **Bhattacharyya Arindam:** Validation, Supervision, Resources, Project administration, Methodology, Formal analysis, Conceptualization. **Ghosh Sumit:** Writing – original draft, Validation, Supervision, Project administration, Methodology, Formal analysis, Data curation, Conceptualization. **Mukherjee Saikat:** Resources, Project administration, Conceptualization. **Ghosh Pronabesh:** Visualization, Validation, Software, Investigation. **Modak Preetam:** Writing – review & editing, Writing – original draft, Visualization, Software, Investigation, Formal analysis, Data curation.

## Declaration of Competing Interest

The authors declare that they have no known competing financial interests or personal relationships that could have appeared to influence the work reported in this paper.

## Data Availability

Data will be made available on request.
